# DNA Replication Stress and Chromosomal Instability: Dangerous Liaisons

**DOI:** 10.3390/genes11060642

**Published:** 2020-06-10

**Authors:** Therese Wilhelm, Maha Said, Valeria Naim

**Affiliations:** 1 CNRS UMR9019 Genome Integrity and Cancers, Université Paris Saclay, Gustave Roussy, 94805 Villejuif, France; therese.wilhelm@curie.fr (T.W.); Maha.SAID@gustaveroussy.fr (M.S.); 2 UMR144 Cell Biology and Cancer, Institut Curie, 75005 Paris, France

**Keywords:** DNA replication stress, chromosomal instability, chromosome segregation, mitosis, cancer, aneuploidy

## Abstract

Chromosomal instability (CIN) is associated with many human diseases, including neurodevelopmental or neurodegenerative conditions, age-related disorders and cancer, and is a key driver for disease initiation and progression. A major source of structural chromosome instability (s-CIN) leading to structural chromosome aberrations is “replication stress”, a condition in which stalled or slowly progressing replication forks interfere with timely and error-free completion of the S phase. On the other hand, mitotic errors that result in chromosome mis-segregation are the cause of numerical chromosome instability (n-CIN) and aneuploidy. In this review, we will discuss recent evidence showing that these two forms of chromosomal instability can be mechanistically interlinked. We first summarize how replication stress causes structural and numerical CIN, focusing on mechanisms such as mitotic rescue of replication stress (MRRS) and centriole disengagement, which prevent or contribute to specific types of structural chromosome aberrations and segregation errors. We describe the main outcomes of segregation errors and how micronucleation and aneuploidy can be the key stimuli promoting inflammation, senescence, or chromothripsis. At the end, we discuss how CIN can reduce cellular fitness and may behave as an anticancer barrier in noncancerous cells or precancerous lesions, whereas it fuels genomic instability in the context of cancer, and how our current knowledge may be exploited for developing cancer therapies.

## 1. Introduction

To maintain a stable genome, at each cell division, a cell must accurately duplicate its genetic material and equally distribute the newly replicated chromosomes in each daughter cell during mitosis. Dysfunctions in one of these processes are the main causes of chromosomal instability (CIN), which is defined as an increased rate of chromosomal changes. As a form of genomic instability, CIN can be manifested as either numerical (i.e., gain and/or loss of whole chromosomes) or structural (e.g., gain, loss and/or rearrangements of parts of chromosomes) termed numerical chromosome instability (n-CIN) and structural chromosome instability (s-CIN), respectively [1]. The source of n-CIN is presumed to lie in mitosis. During mitosis, the cell undergoes a vast reorganization of the cytoskeleton and assembles the mitotic spindle apparatus; the microtubules emanating from the centrosomes connect both spindle poles to the individualized and condensed chromosomes by attaching to the kinetochore structure organized at the centromeric chromatin. The spindle assembly checkpoint (SAC) ensures that each chromosome is correctly aligned and attached to the microtubules emanating from the opposite poles before the two chromatids separate and move towards the opposite poles at anaphase and telophase, after which the cytokinetic furrow divides the two daughter cells. Chromosome segregation errors can occur because of mitotic dysfunctions such as spindle mono- or multipolarity, altered microtubule stability or dynamics, cohesion defects and defective kinetochore assembly or SAC function. In these contexts, major sources of whole chromosome mis-segregation are merotelic attachments [2]. In these cases, one of the two kinetochores is attached to both poles; despite correction mechanisms, the erroneous attachment can persist and cause the mal-oriented chromosome to lag at anaphase, preventing its proper segregation [3].

In the second scenario, the source of s-CIN is distinguished as premitotic, since structural aberrations mainly result from defects in the cellular processes that directly influence chromosome integrity, such as DNA replication and repair. However, these two types of CIN can be interlinked, and they often coexist in cancer cells [4].

Strikingly, one other hallmark of cancer that, similar to CIN, can also be detected in precancerous and cancerous lesions and has a dual role in restraining and promoting cancer development and progression is replication stress [5]. Recent advancements have shown that the co-occurrence of both replication stress and CIN is not based on mere coincidence but that they are highly interdependent.

In the following paragraphs, we analyze the main mechanisms underlying this connection, as well as explain how distinct CIN mechanisms can fuel each other.

## 2. Causes of Replication Stress and Its Role in Chromosomal Instability

“Replication stress” is a broad term that refers to any condition that leads to the hindrance of DNA replication forks or the perturbation of replication dynamics. DNA replication is a highly regulated process that takes place during the S phase of the cell cycle, when the cell duplicates its genetic material. It starts at multiple loci called “origins of replication”, from which replication forks emanate and travel in opposite directions to allow for complete replication of the genome. Replication origins are established (licensed) in late mitosis and the G1 phase before being activated at the G1-S phase transition and fired during the S phase, following a spatially and temporally regulated program [6]. The density of licensed origins and their spatial organization are pivotal for providing the cell with sufficient flexibility to execute the replication program. Indeed, some of the potential origins remain dormant and can be used as backup in case of replication fork stalling [7]. Several exogenous and endogenous threats can lead to replication stress, including DNA lesions or adducts induced by chemical compounds, UV or ionizing radiation, reactive oxygen species (ROS), byproducts of cellular metabolism, nucleotide pool imbalances or a shortage of replication factors [8,9]. Another potential cause of replication stress is linked to the genetic and epigenetic features of specific loci, such as telomeres, centromeres, ribosomal DNA (rDNA) loci or fragile sites, which are intrinsically difficult to replicate due to the presence of repeat sequences that can form secondary structures, the chromatin conformation, origin distribution and replication timing [10,11,12,13,14], as well as to the physiological obstacle presented by transcription [15]. Indeed, when not properly coordinated, the transcription and replication processes can interfere with each other, altering their dynamics, inducing transcription-replication conflicts (TRCs) and promoting the formation of R-loops, three-stranded structures constituted with an RNA-DNA hybrid and a looped-out single-stranded DNA (ssDNA). All these sources of replication stress can slow or stall replication forks and, in the case of sustained replication stress or pathological conditions, induce fork breakage or collapse, resulting in replication-associated DNA double-strand breaks (DSBs) [16].

Replication stress is the main driver of genomic instability in the early preneoplastic stages of tumor development [17,18]. Indeed, recent findings have suggested that the stalling or collapse of DNA replication forks is the prevalent source of DNA damage due to oncogene activation or overexpression (or inactivation of a tumor suppressor), which may contribute to genome instability in the majority of human cancers [5]. Different oncogenes may induce a distinct landscape of genome instability [19], likely reflecting different mechanisms, that, in some cases, overlap, by which they induce replication stress [20]. For instance, RAS overexpression can alter fork speed and cellular metabolism and deplete the nucleotide pool [21], while the deregulated expression of cyclin E can interfere with origin licensing [22] or vice versa, increase origin firing and induce TRCs [23]. A recent study mapped the replication initiation and transcription genome-wide and showed that cyclin E overexpression induces premature entry into the S phase, which leaves insufficient time for transcription to erase origins from genic regions, thus leading to the firing of intragenic origins and TRCs [24]. Similarly, Myc amplification or overexpression can also induce transcription-associated replication stress [24]. In addition, excessive origin firing and deregulated replication dynamics can lead to the exhaustion of nucleotides and reduce the availability of essential replication factors [25,26,27]. The effect of oncogene expression can also depend on the level and duration of the stress, as well as the cell or tissue context [20].

It has become clear from these studies that different sources of stress often converge on a limited pool of vulnerable sites that are particularly sensitive to replication stress and, under these conditions, have the tendency to undergo breakage during mitosis [13]. These sites, called common fragile sites (CFSs), have been known to cytogeneticists since the 1980s, when—while studying the cytological expression of the fragile X chromosome associated with mental retardation—researchers observed recurrent breaks in mitotic chromosomes in lymphocytes cultured in folate-deprived medium or exposed to low doses of aphidicolin (APH), an inhibitor of DNA polymerase α [28,29]. Interestingly, it has been shown that CFSs are also involved in DNA damage induction following oncogene activation in early preneoplastic stages of tumor development, indicating that the induction of their instability may be a key step in the tumorigenic process [17,18,30,31]. In addition to oncogene activation, the loss-of-function of caretaker genes that encode proteins involved in the maintenance of genome stability also enhances their instability [32,33,34,35]. The study of the mechanisms underlying CFS instability has shed light on the important link between replication stress and CIN [36]. They have shown that these loci often remain incompletely replicated during the S phase, leading to the persistence of replication intermediates until G2 and mitosis, when they are processed by specific pathways that, by resolving these structures, rescue cells from replication stress and allow proper sister chromatid separation and mitotic cell division [37]. Hereafter, we discuss how replication stress can impact the mitotic process and result in both structural and numerical CIN, acting to restrain proliferation or promote genetic diversification during tumor evolution. This connection between replication stress and CIN may be key to understanding the pathogenetic mechanisms involved in chromosomal instability and cancer-prone syndromes, such as ataxia telangiectasia (AT), familial cutaneous telangiectasia and cancer syndrome, Seckel syndrome, Bloom syndrome or Fanconi anemia, as well as to understanding the role of CIN in cancer [38,39,40,41,42,43].

### 2.1. Mechanisms Protecting against Replication Stress and Under-Replicated DNA during Mitosis

To protect the genetic material from endogenous or exogenous assaults, eukaryotic cells have evolved the DNA damage response (DDR), a complex signal transduction pathway in which DNA damage is detected and activates the core DDR transducer kinases, the ataxia telangiectasia-mutated (ATM), ATM and Rad3-related (ATR) and DNA-PKcs to orchestrate the cell response to specific DNA lesions and alterations to the DNA structure and promote their repair [44]. The cellular response to replication stress is primarily controlled by the ATR kinase, while ATM and DNA-PK most prominently respond to DSBs and contribute to ATR activation under specific damaging conditions or act as a backup [45,46,47,48]. ATR is activated by DNA damage or stress that impedes replication fork progression and leads to the generation of ssDNA. The stepwise activation of ATR signaling and phosphorylation of its main downstream effector CHK1 upon different levels of replication stress or the formation of specific structures may elicit the different responses necessary to stabilize or resume replication fork function, regulate origin firing, delay cell cycle progression and repair any damage before the cell enters mitosis [45,49].

Under replication stress conditions, the uncoupling of DNA polymerase and MCM helicase at stalled replication forks results in the formation of ssDNA that is instantly protected by an RPA coating, recruiting ATR and its partner ATRIP, which activate downstream effectors to stabilize the forks and activate the checkpoint [50]. ATR signaling is allosterically stimulated by TOPBP1 [51], which may serve to amplify ATR signaling [45]; strikingly, a study showed that the expression of a TOPBP1 protein mutated in the ATR-activating domain slowed the rate of DNA replication elongation while increasing the origin firing in the S phase, providing evidence for the implication of ATR in the spatiotemporal regulation of DNA replication and the response to replication stress [16,52]. The maintenance of the spatiotemporal program of replication by ATR safeguards stalled replication forks from breakage and prevents replication catastrophe. ATR confers this protection by suppressing the excessive firing of replication origins that can otherwise lead to the exhaustion of the rate-limiting pool of RPA and conversion of ssDNA at stalled replication forks to DNA double-strand breaks (DSBs) in the S phase, a serious threat to genome stability [27].

Additionally, under unperturbed conditions, ATR and CHK1 are activated to limit dormant origin firing in actively replicating sites. By stabilizing the PP1 phosphatase and RIF1 interaction, ATR and CHK1 prevent the phosphorylation of RIF1 and inhibit the assembly of the CMG helicase at replication origins [53]. Interestingly, studies on the activation of ATR-CHK1 in unperturbed conditions led to the identification of a distinctive allosteric activator, Ewing’s tumor-associated antigen 1 (ETAA1) [16,54,55,56]. Although it is still unclear how the activation of ATR by ETAA1 is triggered in the unperturbed S phase, it is thought that the transient ssDNA generated by ongoing DNA replication triggers this activation that continues throughout the S phase to the transition point with G2. It has also been shown that the phosphorylation of FOXM1, which is dependent on cyclin-dependent kinase 1 (CDK1) activation, occurs at exactly this S/G2 transition, promoting the execution of a mitotic transcriptional program. Accordingly, the delay in the activation of CDK1 itself is caused by CHK1, ensuring that the cell does not progress to G2 prior to completion of DNA replication in the S phase [57]. Similarly, a recent study by the Lindqvist laboratory has shown that active DNA replication restricts the activation of CDK1 and PLK1 mitotic kinases through CHK1 [58]. Another recent study in yeast has shown that the basal activation of the Mec1-Rad53 (the functional homologs of ATR-CHK1) pathway results from the spontaneous replication stress triggered by an insufficient level of dNTPs in the early S phase that contributes to the coordination of the origin activity with dNTP synthesis, a function conserved in mammals [47,59,60]. Interestingly, however, the essential function of Mec1-Rad53 is linked to the maintenance of replication fork integrity, which prevents cells bearing under-replicated and damaged chromosomes from progressing to mitosis [59]. The stabilization of the replication fork protects the stalled fork from degradation and maintains its competence to resume DNA synthesis upon removal or bypass of the replication obstacle [45,61]. If the stalled fork cannot be rescued by an incoming fork and cannot resume, then it can undergo fork collapse, a process that involves the generation of a DSB at the fork. A DSB end can be an intermediate structure that enables a recombination-mediated restart or can be the result of the active cleavage of terminally arrested or remodeled forks. Proteomic studies of proteins associated with replication forks in the absence of ATR have shown that fork collapse does not involve the dissociation of the replisome but a dynamic change in its composition [62]. For a detailed overview of the mechanisms involved in replication-coupled repair and the pathways involved in the protection, remodeling and processing of stalled replication forks, the reader is directed to the following reviews [16,63]. Homologous recombination (HR) factors such as RAD51, BRCA1 and BRCA2 and members of the FA pathway, disabled in the chromosome instability and cancer predisposition syndrome Fanconi anemia, are central regulators of replication stress tolerance through their functions in replication fork protection, which are distinct from their canonical roles in the HR-mediated repair of collapsed forks [63]. Following replication stress, the recruitment of the FA protein FANCD2 and of TOPBP1 to stalled forks is promoted by the microRNA pathway enzymes Dicer and Drosha, which foster the activation of the ATR-dependent S phase checkpoint and limit cells with under-replicated DNA from proceeding into mitosis [64].

These findings are in agreement with those of a previous study, showing that, in the presence of ATR and CHK1, low levels of under-replicated DNA were able to escape surveillance and continue into mitosis; furthermore, this portion of “leaked” under-replicated DNA was increased after ATR depletion [65]. It is unclear whether this leakage underlies a low level of active forks or terminally arrested forks that do not signal to the checkpoint. Indeed, several studies have shown that replication completion at some loci in the genome, notably CFSs, can be delayed after the S phase, particularly after replication stress; thus, replication intermediates persist until G2 and mitosis [37,66]. A recent study from Lafarga and collaborators identified the RNA-binding protein TIAR as a novel component of a G2/M checkpoint that prevents cells with under-replicated or damaged DNA from entering mitosis. TIAR accumulates in G2/M transition granules (GMGs) during an unperturbed cell cycle and upon the induction of replication stress and retains CDK1, attenuating its activity. Interestingly, GMGs are enriched with proteins recruited to stalled replication forks and with components of the transcription machinery, suggesting that local transcription activity or TRCs may serve as signals that activate a G2/M checkpoint [67].

The Cimprich laboratory has shown that the orientation of TRCs determines distinct DNA damage responses, with head-on collisions between transcription and replication machineries promoting R-loop formation and ATR activation, and co-directional collisions promoting R-loop resolution and leading to ATM activation, likely as a consequence of DSB formation, at R-loop-prone sequences [68].

Previous studies have also suggested a role for ATM in the replication stress response, where ATM with the MRE11–RAD50–NBS1 (MRN) complex promotes the HR-mediated recovery of stalled or collapsed replication forks [9,69]. ATM has also been observed to cooperate with downstream helicases, WRN and BLM, in the response to replication fork stalling [70,71]. Recently, and with a similar cellular setup, Fugger et al. showed that, following hydroxyurea (HU)-induced replication stress, F-box DNA helicase 1 (FBH1), which is implicated in replication fork regression, activates ATM signaling in response to fork stalling independent of DSB formation. Interestingly, they also showed that, following FBH1 depletion under the HU treatment conditions, the cells entered mitosis sooner than their control counterparts, pointing towards a role for FBH1 in G2/M checkpoint control, which can limit the genomic regions with inactivated or damaged forks from progressing into mitosis [72]. In addition, in a role independent of DNA damage, ATM has been recently found to cooperate with p53 in the mitotic surveillance pathway in nontransformed human cells. It has been reported that the relocalization of p53 from the cytoplasm to mitotic centrosomes occurs only following its phosphorylation by ATM. The authors also showed that inhibition of p53 centrosomal localization, or even acute p53 depletion, led to centrosome fragmentation and, eventually, to cell death, highlighting another mechanism by which genome stability is preserved before the next cell cycle is initiated [73]. Similarly, aside from its role in the replication stress response, ATR has an independent role in preventing CIN in mitosis, as was recently described. ATR was shown to be recruited to centromeres in an R-loop-dependent manner, where it activates Aurora B to promote faithful microtubule binding and subsequent chromosome segregation [74]. The tight coordination between the DDR pathways and cell cycle checkpoints throughout the S phase and mitosis is thus key to preventing genome instability [66].

### 2.2. Replication Stress Links Structural and Numerical Chromosomal Instability

The first hints about a connection between the premitotic and mitotic origins of CIN came from the identification of the role of factors known to be involved in DNA repair pathways, such as Bloom syndrome helicase (BLM) and FA proteins, during mitosis [75,76,77,78]. The FA pathway, best known for its function in the repair of interstrand crosslinks, toxic lesions that prevent DNA strand separation and block replication and transcription, also plays a major role in the response to replication stress and the maintenance of CFS stability [34,79,80]. By looking at the localization pattern and behavior of FANCD2, a key member of the FA pathway, it was determined that part of the chromosomal instability characteristic of FA-deficient cells was due to defects in chromosome segregation during mitosis, particularly upon replication stress [78]. FANCD2 was shown to localize to discrete sites on mitotic chromosomes after the induction of replication stress with low doses of APH, a common inducer of CFS expression [29]. Indeed, FANCD2 was localized to APH-induced gaps and breaks on mitotic chromosomes, where it colocalized with γ-H2AX, a marker of DBSs [76,77]. In addition, by cooperating with the helicase BLM, FANCD2 was shown to promote the resolution of ultrafine bridges (UFBs) [77], thin DAPI-negative DNA threads that evolve from joint DNA molecules, arising from replication or recombination intermediates that persist until mitosis and are resolved during anaphase and telophase [81,82,83,84].

### 2.3. The Ultrafine Bridges (UFBs): An Overlooked Form of Anaphase Bridges

In 2007, a study from Erich Nigg’s laboratory led to the identification of the Plk1-interacting checkpoint “helicase” (PICH), an SNF2 family ATPase that was found to decorate centromeric thread-like structures connecting sister kinetochores from metaphase to anaphase [85]. In that same year, by investigating the BLM helicase function in chromosome segregation, Ian Hickson’s laboratory showed that BLM and PICH localized to the same DAPI-negative DNA bridges during anaphase [75].

These two studies defined a peculiar class of anaphase bridges, termed “UFB”, which are not stained by conventional DNA dyes, such as DAPI or Hoechst, and are devoid of histones, contrary to bulky chromatin bridges. While bulky anaphase bridges result from chromosome/chromatid fusion, UFBs arise from persistent sister chromatid DNA entanglement.

**Figure 1:**
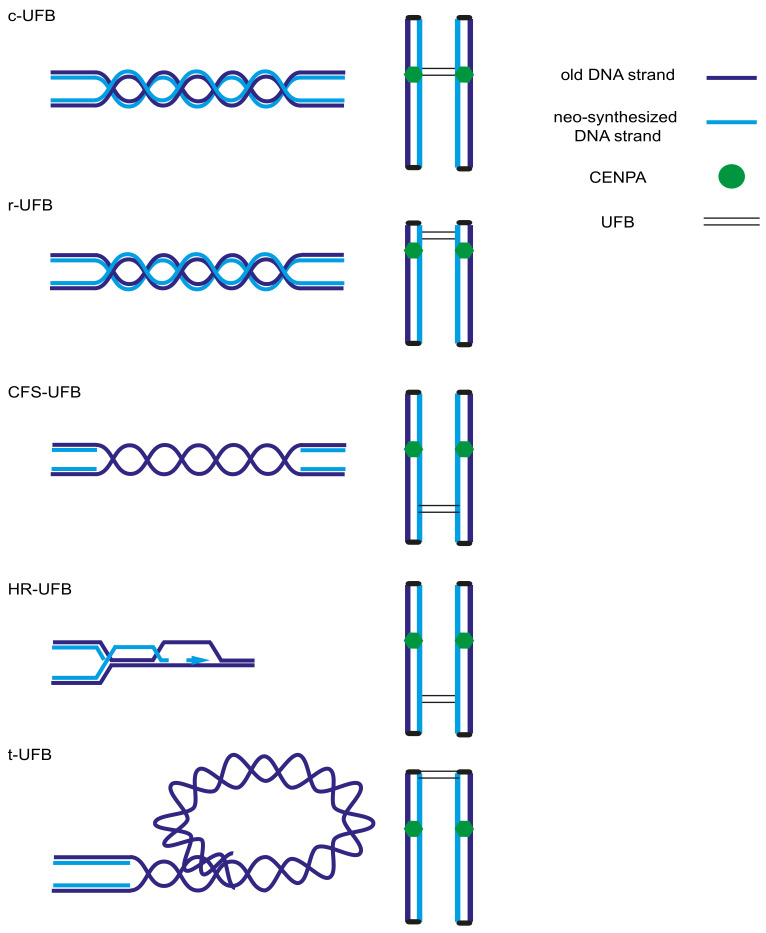
The different types of ultrafine bridges (UFBs).

Today, at least five different subclasses of UFBs are known based on their loci of origin and/or underlying DNA structures (Figure 1), as discussed below: centromeric (c-UFBs), ribosomal (r-UFBs), common fragile site (CFS-UFBs), telomeric (t-UFBs) and homologous recombination UFBs (HR-UFBs). c-UFBs and r-UFBs arise from the formation of double-stranded DNA catenanes (i.e., completely replicated intertwined DNA) driven by the repetitive nature and sequence content of centromeres and rDNA loci. On the other hand, CFS-UFBs and t-UFBs are mainly derived from under-replicated DNA or late-replication intermediates, and the newly identified HR-UFBs are derived from persisting recombination intermediates [82,83,86]. A complex composed of BLM and its binding partners, topoisomerase IIIα and RecQ-mediated genome instability protein 1 (RMI1) and RMI2 (BTRR), together with PICH (DNA translocase) and topoisomerase II, act by untangling structures underlying different types of UFBs, facilitated by additional factors such as the DNA translocase FANCM, RIF1 and TOPBP1, which are recruited to a subset of UFBs [87,88,89,90].

c-UFBs are the most prevalent type of UFBs that form under physiological conditions as a consequence of the late decatenation and disjunction of centromeric sister chromatid DNA [91,92]. Topoisomerase II inhibition increases UFB frequency, indicating its role in the decatenation of dsDNA at c-UFBs. Similar to its role in c-UFBs, topoisomerase II is recruited by PICH to r-UFBs. It is believed that the transcription that overlaps mitotic chromosome condensation and hinders topoisomerase II activity drives r-UFBs [86,93].

In contrast to the c- and r-UFBs observed under normal conditions, CFS-UFBs were discovered following replication stress induction by APH. Under replication stress conditions, under-replicated DNA persists into mitosis, forming CFS-UFBs, and FANCD2 and FANCI mark sister chromatid interlinked structures at their extremities, possibly to orchestrate their resolution by other factors [37,76,77]. RPA binding to ssDNA on the CFS-UFBs and the absence of an effect of topoisomerase II inhibition on them reinforces the idea that CFS-UFBs are generated because of under-replicated DNA or unresolved replication intermediates rather than dsDNA catenanes [76,81,94].

On the other hand, HR-UFBs arise as a consequence of double-strand break repair and the subsequent formation of stable Holliday junctions. Persistent HR-UFBs can lead to segregation defects in cells defective for recombination intermediate resolution [95]. HR-UFBs were defined by the association of BLM and RPA with FANCD2 negative-UFBs in early anaphase. In late anaphase, HR-UFBs are converted by PICH and BLM into RPA-bound ssDNA that is susceptible to breaks [96]. Camptothecin, a topoisomerase I inhibitor, increases HR-UFB levels, indicating that DSBs are required for the formation of the recombination intermediates that induce HR-UFBs [37,83].

T-UFBs seem to possess some properties of CFS-UFBs, r-UFBs and c-UFBs. T-UFBs can be induced by replication stress (similar to CFS-UFBs), which affects the completion of replication of difficult-to-replicate loci. Impaired telomere replication in Werner’s syndrome (WS)-deficient cells induces the recruitment of the BTR complex to facilitate t-UFB resolution [97]. T-UFBs can also be induced by the downregulation of telomeric repeat binding factor 1 (TRF1), which facilitates telomere replication and protects telomeres from end-to-end fusion, or by the overexpression of TRF2 [97,98]. Surprisingly, t-UFBs are also induced by topoisomerase II inhibition, similar to r-UFBs and c-UFBs, indicating that t-UFBs may comprise both unresolved replication intermediates and dsDNA catenanes [99].

Finally, another kind of UFB, which depends on RAD51 and is prevented from forming by 53BP1, has been observed in cancer cells and leads to a rupture of the sister chromatid axes followed by chromosome bridging, resulting in gross chromosomal rearrangements [100]. This phenomenon is distinct from breakage-fusion-breakage cycles, in which anaphase bridges generated by chromosome/chromatid fusions undergo breakage and then rejoining events [101,102].

Diagram depicting the different types of UFBs. UFBs that arise from persistent double-stranded catenanes are formed at centromeres (c-UFBs) or originate from rDNA loci (r-UFBs). Under-replicated DNA or unresolved replication intermediates induce common fragile site UFBs (CFS-UFBs). Recombination intermediates as Holliday junctions drive homologous recombination UFBs (HR-UFBs). Unresolved replication intermediates and double-stranded catenanes induce telomeric UFBs (t-UFBs) (cc-by [83]).

When UFBs are not properly resolved before anaphase, the sister chromatids remain interlinked, which can lead to chromosome nondisjunction or breakage, visible as micronuclei containing whole chromosomes and/or chromosome fragments. In addition, when the DNA comprising the UFB is not properly protected or resolved during anaphase or telophase, it may be ruptured by the end of mitosis [37,78]. These findings thus showed that perturbed DNA replication or the failure to respond to replication stress can have direct consequences on the mitotic process by preventing replication completion or accurate repair of specific difficult-to-replicate regions. In addition, replication stress was shown to induce numerical aneuploidy after the first mitotic division in the primary fibroblasts of FA patients, demonstrating the link between replication stress and chromosome mis-segregation [77]. The formation of UFBs or bulky chromosome bridges in FA-deficient hematopoietic cells was also reported to induce cytokinesis failure and lead to binucleation [87]. In agreement with these findings, DNA lesions and mitotic aberrations following oncogene-induced replication stress have also been found to induce cytokinesis failure and tetraploidization, leading to whole genome doubling [103], a common step in tumor development [104].

### 2.4. Mitotic Rescue from Replication Stress

Further insights into the link between replication stress and CIN came from the discovery of the replication stress protection conferred by specialized polymerases that are able to replicate across noncanonical DNA and AT-rich sequences at difficult-to-replicate regions [105,106]. Notably, it was shown that the translesion synthesis DNA polymerase eta is recruited to specific CFSs during the S phase and promotes their timely replication [107]. Pol eta deficiency causes delayed replication completion, as shown by the incorporation of the thymidine analogue EdU in late G2 and mitotic cells and the persistence of under-replicated DNA at CFSs on mitotic chromosomes marked by FANCD2, leading to the transmission of DNA damage to daughter cells, shielded in 53BP1 nuclear bodies [107,108,109]. Recruitment of pol eta to replication forks during unchallenged replication or mild replication stress was later shown to be regulated by pol η SUMOylation by the PIAS1 SUMO ligase and RAD18, independent of the RAD18-mediated PCNA ubiquitylation that alternatively regulates the recruitment of pol eta to UV-induced DNA lesions [110].

This work confirmed the long-standing observation indicating the late replication timing of CFSs [111] and supporting the idea that, despite a functional checkpoint, some replication intermediates or under-replicated DNA structures may be checkpoint-blind or tolerated, such that the cells can enter mitosis with a fraction of its genome not completely replicated [112]. Intense work from various laboratories has substantiated this concept and showed that under-replicated or unresolved DNA structures are subjected to active processing and resolution in mitosis, allowing mitotic rescue from replication stress (MRRS) and faithful chromosome segregation [37]. Notably, late replication intermediates are processed by components of the structure-specific endonucleases SLX4, XPF-ERCC1 and MUS81-EME1 [113,114,115,116]. Importantly, by targeting under-replicated DNA, these endonucleases promote the formation of chromosome breaks typically observed at CFSs in metaphase; however, these breaks allow sister chromatid separation, preventing chromosome segregation defects and mitotic catastrophe. Active processing by endonucleases is necessary to complete CFS replication in mitosis by a mechanism akin to break-induced replication (BIR), which is dependent on POLD3 and RAD52 [117,118]. This mechanism is also involved in rescuing replication stress at telomeres during mitosis [119,120,121,122]. Furthermore, recent studies have shown that the ubiquitin ligase TRAIP promotes replisome disassembly to drive mitotic DNA synthesis [123,124]. While this mechanism allows the cell to complete replication and to limit chromosome segregation defects, it may also be mutagenic and lead to chromosomal rearrangements [125]. Therefore, it is likely that the capability of a cell to respond to replication stress and the outcome of the mitotic rescue from replication stress are dependent on the magnitude of stress and the cellular context.

A subsequent study confirmed that replication stress links n-CIN and s-CIN in colorectal cancer cells [126]. In chromosomally unstable cancer cells, candidate suppressors of replication stress, such as PIGN, MEX3C or ZNF516, are often inactivated. When the authors silenced these genes in chromosomally stable cells, replication stress increased the number of structural chromosome aberrations and coincided with numerical aneuploidy, as revealed by the deviation from the modal number of centromere signals of chromosomes 2 and 15. Furthermore, APH treatment in chromosomally stable cells similarly affected the segregation of chromosomes 2 and 15, which led the authors to suggest that replication stress can indeed induce n-CIN.

Interestingly, another study reported that homologous recombination (HR)-deficient cells are characterized by endogenous replication stress that can have both local and global consequences on chromosome segregation [44]. It showed that endogenous replication stress elicited by HR deficiency or treatment with low doses of HU or APH altered the centrosome number, leading to multipolar mitoses and global chromosome mis-segregation. Therefore, replication stress can impact not only the segregation of under-replicated or structurally aberrant chromosomes but, also, the fidelity of the mitotic process.

## 3. How Replication Stress Affects Mitotic Fidelity

The fidelity of the mitotic process is primarily surveilled by the spindle assembly checkpoint (SAC) that, together with other error correction mechanisms, prevents the segregation of unattached or incorrectly attached chromosomes in mitosis and delays mitotic exit [127,128]. Loss-of-function mutations in SAC proteins are rare in tumors, most likely because they are incompatible with cell survival [129,130]. Their operational necessity is nicely demonstrated in experiments performed with CENPE inhibitors, which prevent correct chromosome attachment, used in conjunction with SAC inhibition, which led to a high level of aneuploidy and cell death [131]. These data suggest that even if a subpopulation of tumor cells might bear SAC mutations, they do not seem to be the main contributors to numerical chromosomal instability in sporadic cancers. Here, we summarize evidence of replication stress-dependent mechanisms that can activate and/or attenuate the SAC and may be responsible for the occurrence of numerical aneuploidy in cancer cells.

### 3.1. Replication Stress and Spindle Assembly Checkpoint (SAC)

Each sister chromatid builds a multilayered proteinaceous structure onto centromeric DNA that is locally restrained to zones of CENPA nucleosomes deposited in the previous G1 phase [132]. This multilayered structure, the kinetochore, is captured by microtubules emanating from the centrosomes. The kinetochore of one chromatid will attach to one spindle pole, whereas the sister kinetochore of the other chromatid attaches to the opposing spindle pole. In this “amphitelic” configuration, the tension between the sister kinetochores that is created by the pulling forces of the opposing centrosomal microtubules creates sister kinetochore distancing. This tension is needed to stabilize end-on attachments (i.e., attachments to microtubule plus ends) and satisfy the SAC, which permits progression into anaphase [127]. Mad1 and Bub1 occupy nonattached kinetochores, and then, the mitotic checkpoint complex (MCC), consisting of Mad2, Bub3, BubR1 and Cdc20, is assembled. Next, Mad2/BubR1-dependent signaling prevents the degradation of cyclin B1 and securin, the action of which is normally triggered by the anaphase-promoting complex/cyclosome (APC/C). Sustained high cyclin B1 activity prevents mitotic exit, and high securin levels inhibit the activity of separase, which is necessary to cleave the remaining centromeric cohesion that holds both sister chromatids together [127]. An Aurora B-dependent error correction pathway that detects and corrects incorrectly attached kinetochores influences the SAC. Distinct populations of this kinase, located at the centromere and kinetochore region [133], regulate its activity toward downstream targets such as Ndc80 and Dsn1 that then facilitate the detachment of incorrectly attached microtubules. Kinetochores that are not end-on attached recruit Mps1 kinase, which then wires this error correction to the SAC by recruiting Mad2 and BubR1 [128].

The ability of the SAC to detect and respond to replication stress/DNA damage in mitosis appears to be dependent on the severity of the stress, and, based on the experimental settings, different observations were made. Research conducted to study kinetochore detachment, kinetochore tension or asymmetric DNA strand segregation induced mitosis with unreplicated genomes (MUGs). This condition can be achieved by high doses of HU in combination with caffeine, cyclin A overexpression, HU-only treatment or by silencing the origin of replication protein DUP [134,135,136,137]. The complete absence of a sister chromatid may be considered an extreme case of replication stress/acute under-replication; yet, despite prolonged mitotic timing, these cells with just one kinetochore eventually managed to proceed into anaphase. Nocodazole (a tubulin-binding agent that interferes with microtubule polymerization) did still potently arrest these cells in mitosis, which shows that the SAC was not compromised [135], suggesting that the mitotic checkpoint is partially blind and that acute replication stress can be transmitted throughout mitosis.

Recently, it was shown that strong replication stress in primary IMR90 fibroblasts leads to p53-dependent interphase cell cycle arrest. The very same treatment in p53-deficient IMR90 E6E7 cells leads to sustained Mps1-dependent mitotic arrest and mitotic death, which suggests that severe under-replication that escapes interphase surveillance can be sensed and retained in mitosis [138]. In the latter case, the replication stress induced by APH or HU led to a severe delay in interphase. The more pronounced the interphase delay, the more it correlated with permanent mitotic arrest, which insinuates that a specific threshold of under-replication and/or damage has to be reached before cells are permanently arrested and die in mitosis. Mitotic cell death occurs via two independent pathways, one involving apoptosis by BAX/BAK (Type 1) and another depending on telomere deprotection and activation of an Aurora B-, TRF2- and ATM-dependent DNA damage response at telomeres (Type 2) [138].

Whereas the first studies show that even completely unreplicated cells can enter anaphase and exit mitosis, the second scenario shows that a high stress level leads to mitotic cell death, which seem like contradictory findings. We envision that, in the first scenario, the complete absence of one chromatid allows microtubules to attach to the remaining chromatid (merotelic attachment), and the checkpoint is satisfied after prolonged mitosis. This theory fits with the proposition that interkinetochore distance and tension are not needed to satisfy the checkpoint [135,139]. In the second scenario, cells might have partially replicated genomes, and microtubules may try to catch both sister kinetochores. It is possible that the centromere/kinetochore structure is somehow compromised here, leading to a defective establishment of tension. Even though it does not directly activate the SAC, an absence of tension may activate the Aurora B-mediated error correction [140], which would fuel continuous Mad2/BubR1-dependent SAC signaling from unattached kinetochores, delaying anaphase onset and allowing apoptotic cell death and telomere deprotection pathways.

Recent findings in yeast tubulin mutants with increased sensitivity to the DNA replication stress/DNA-damaging agents HU and 4-nitroquinoline 1-oxide (NQO) substantiate the previous suggestion. These mutants were shown to arrest in mitosis after treatments with high doses of HU followed by release in a normal medium. This arrest was not due to the classical activation of the SAC but to an Aurora B-dependent tension checkpoint, and the authors suggested that the observed phenotype may arise because tubulin mutants fail to detect some DNA damage in interphase that is carried over to mitosis. Such damage may cause tension defects, as the centromere, even if fully replicated, may contain centromeric lesions that compromise kinetochore assembly and kinetochore microtubule attachment [141].

As nonreplicated chromosomes hardly arise in a physiological context and strong HU/APH-induced replication stress leads to mitotic death, we believe that the most severe threat comes from low replication stress that is not or only partially sensed in mitosis. We hypothesized that, under these circumstances, cells survive or at least exit mitosis, which we showed to be the case for noncancerous RPE1 cells challenged by low doses of APH. As RPE1 cells are supposed to be proficient in all checkpoints, we concluded that low replication stress remained below the threshold of detection in mitosis [142].

An interesting study in yeast provides one possible explanation of how a sustainable amount of replication stress can attenuate/shut off SAC activation and, therefore, does not interfere with mitotic progression [143]. SUMO-targeted ubiquitin ligases (STUbls), such as Slx5/Slx8 (RNF4), regulate the ubiquitination of SUMOylated proteins and, therefore, modulate their activity. The authors studied the SUMO proteome in a Mcm10-deficient background that mimics replication stress and is synthetically sick with a Slx5/Slx8 deletion and identified members of the chromosome passenger complex (CPC), a complex consisting of Ipl1 (Aurora B), Sli15 (Incenp), Nbl1 (Borealin) and Bir1 (Survivin) that controls several aspects of mitosis. The SUMOylated CPC proteins Sli15 and Bir1 were shown to be destabilized in a Slx5/Slx8-dependent manner in the presence of replication stress. As a final conclusion, it was suggested that replication stress partially activates the Mad1/2-dependent SAC and that the Slx5/Slx8 complex relieves mitotic arrest [143].

Intriguingly, another target regulated by RNF4 (Slx5/Slx8) is FANCD2/FANCI. Extraction of the FANCD2/I complex from damaged foci was shown to depend on RNF4-dependent polyubiquitination and consequent proteasomal degradation. Such timely turnover at damaged sites seems to be required to guarantee downstream repair pathways to correctly take over [144]. FANC-deficient cells show defects/leaks in the SAC, which might partially explain their numerical aneuploidy phenotype [145]. We could speculate that FANC proteins are recruited to kinetochores after replication stress to coordinate mitotic progression based on the amount/severity of stress and that such coordination/surveillance is absent in FANC-deficient cells, engendering a leaky SAC. However, more studies are needed to investigate this hypothesis, as FANC proteins have not been found to date at the kinetochore after APH treatment [113].

Another interesting study conducted in yeast found that DNA damage can lead to an “inflammation zone” at the chromosome, and when this zone is sufficiently close to a centromere, it can lead to epigenetic centromere changes; these changes are premitotically detected by the SAC (in this case Mad2), which then overlaps with the canonical Mec1/Tel1 (ATR/ATM) checkpoint and delays G2. This finding indicates an involvement of the SAC in cell cycle delay even earlier than originally anticipated and not only prior to anaphase [146]. Finally, this outcome might be attributed to the interdependency of Rad53 (CHK1) and Mad2 after replication stress, as it has been shown that origin firing is compromised in yeast Mad2 mutants in a Rad53 mutant background. Mad2 actively promotes S phase cyclin translation that then influences replication origin firing [147].

In conclusion, we suggest that low replication stress is the most severe threat to genome integrity, as mitotic checkpoints are either blind to detecting it or can actively override it. In the end, such a mitotic exit can be accompanied by structural and numerical chromosomal aberrations [126,142,148].

### 3.2. Replication Stress and Spindle Microtubules

The previously discussed checkpoint in mitosis is strongly interdependent with microtubule dynamics, as indicated by the finding that compromised microtubule stability and/or turnover possibly lead to erroneous kinetochore-microtubule attachments [149].

Microtubule stability is posttranscriptionally regulated and depends on the stability of its own mRNA to ensure that microtubules can react promptly to diverse insults [150]. This mechanism depends on the availability of tubulin monomers, which negatively regulate mature spliced mRNA [151]. It could be that the extent of DNA damage that is transmitted into mitosis is wired to the dynamic instability of the microtubules that can promptly react to these changes. When this adaptation is compromised, as might be the case for the tubulin mutants, viability is decreased [141].

It has been shown that DNA repair factors can be recruited to mitotic spindles [145,152,153] or the main microtubule organizing centers, the centrosomes [145,154,155,156,157,158,159]. However, it remains difficult to determine whether the presence of these proteins in mitosis has an actual role in regulating microtubule stability, as experiments using protein depletion make it complicated to discern the role of these proteins in interphase and mitosis. Factors such as 53BP1 or RNF8 that are phosphorylated and therefore inactivated by mitotic kinases need to remain silent, and their reactivation in mitosis can lead to Aurora B-mediated telomere fusion and aneuploidy [160]. Notwithstanding the partial activation of the DNA damage response, at least the initial steps, such as γ-H2AX and ATM autophosphorylation, proceed in mitotic cells [161]. The idea is that assuring a timely passage through mitosis is prioritized over DNA repair and that DNA repair in mitosis would be anyways more deleterious than beneficial. It is also suggested that the sites of DNA damage are marked by DNA repair factors to guarantee faithful repair in the following interphase [108,161].

Our own data from experiments with low-dose replication stress confirmed that mitotic microtubule stability is altered when cells are exposed to APH in the previous interphase [142]. As we detected a dose-dependent increase in metaphase breaks, we speculated that mitotic damage leads to microtubule stabilization under these conditions. Centriole disengagement was used as a proxy for this stabilization, as we suggested that the dominant problem caused by microtubule stabilization was premature disengagement of the centrioles. We rescued centriole disengagement with ATR inhibition [142], suggesting that DNA replication stress/DNA damage activates ATR and that ATR signaling stabilizes microtubules. Other studies have reported that mild induced replication stress or intrinsic replication stress in CIN^+^ cells alter microtubule dynamics by increasing microtubule plus-end growth. Merotely and chromosome mis-segregation occurred here in the absence of spindle multipolarity and was entirely due to microtubule alterations [148].

Another study by Bakhoum and colleagues confirmed the abovementioned findings and provided a possible explanation for how DNA damage impacts microtubule stability. This is one of the few reports that observed prompt microtubule dynamicity changes after DNA insults within the same mitosis. The dominant chromosome segregation error was whole chromosome lagging due to increased microtubule stability. Slow decaying kinetochore microtubules were the ones affected by DNA damage, suggesting that the attachment to kinetochores is specifically stabilized. γ-H2AX, ATM and CHK2 signaling were implicated in stabilizing microtubule attachment after DNA damage. The downstream actors that were hyperphosphorylated under these conditions and directly interfered with microtubule stability were Aurora A and PLK1. In conclusion, a DNA damage-signaling cascade with ATM-CHK2-PLK1-Aurora A leads to microtubule stabilization and lagging chromosomes and connects the DNA damage response to the cytoskeleton [162].

Centrosomes are the main microtubule-nucleating center in cells and recruit DNA repair factors, which, in turn, can influence microtubule nucleation rates. The Bastian laboratory showed that chromosomally instable (CIN^+^) colorectal cancer (CRC) cells have increased microtubule plus-end growth, compared to nontumorous RPE1 and CIN^−^ CRC cells, as shown by the microtubule plus-end protein EB3 tracking in space and time. Aurora A and CHK2 were identified as the main regulators of microtubule assembly in CIN^+^ CRC cells. CHK2-dependent phosphorylation of BRCA1 at the centrosomes limits the centrosomal pool of Aurora A, which complies with normal microtubule assembly rates generated by the centrosomes. Loss of CHK2 or Aurora A overexpression led to excessive accumulation of Aurora A on centrosomes and increased the microtubule assembly rates [163], suggesting that the CHK2-BRCA1 axis attenuates Aurora A activity at centrosomes to prevent chromosome mis-segregation [164]. Altogether, this finding provides another mechanism by which DDR factors in mitosis influences microtubule stability and dynamics and connects DNA insults to the cytoskeleton.

### 3.3. Replication Stress and Centrosomes

Now, we briefly discuss how centrosomes duplicate and how replication stress/DNA damage interferes with this process. Centrosome duplication is a semi-conservative process that has to be precisely synchronized with DNA replication in order to enter mitosis with two centrosomes, each containing two centrioles [165]. That this vulnerable process is already aberrant/misregulated in precancerous lesions was recently shown, and many cancer cells show centrosome overamplification [166]. As multiple centrosomes might jeopardize canonical bipolar segregation, cells overcome this issue by clustering supernumerary centrosomes before anaphase [167,168]. In G1, centrosomes consist of one centriole each. Procentriole formation is initiated at G1/S and elongation of the procentriole, and distancing from the mother centriole occurs in S, G2 and mitosis [169]. The perpendicular establishment of the daughter is suggested to create an intrinsic block to reduplication. Initially, it was proposed that PLK1 in early mitosis and separase later in mitosis drive centriole separation, as PLK1 inhibition partially delays disengagement [170]. Distancing of the daughter centrioles within one centrosome is a PLK1-dependent process, which seems to depend on pericentriolar material (PCM) maturation. Increased PLK1-dependent PCM maturation accompanied by the distancing of both centrioles within centrosomes is believed to be the driving event of canonical centriole disengagement at the mitotic exit [171].

Pioneering work from Sibon et al. established the link between checkpoint-proficient mutants that presented under-replicated DNA in mitosis and consequent mitotic failures. This was based on initial observations of interphase-checkpoint-deficient *Drosophila* embryos, in which mitotic spindle establishment was affected by the lack of components of the PCM, leading to anastral spindles. This so-called “centrosome inactivation pathway” was considered a backup mechanism in case replication and/or repair processes were not completed by mitotic entry. In the end, this mechanism would prevent the propagation of genetic instability in the next generation [172]. DmChk2 checkpoint kinase is essential for this inactivation [173], and together with the observation that several components of the checkpoint/repair machinery are frequently detected at centrosomes, such as BRCA1 [174], p53 [175], BRCA2 and CHK1 [158], it was suggested that centrosomes are actively implicated in the response to DNA damage in mitosis.

In mammalian systems, hampered DNA replication/DNA damage seems to lead to slightly different phenotypes. Here, the centrosome and its components are present and not “inactivated”; however, the integrity of the PCM is affected, leading to centrosome splitting and the PCM containing only one centriole. High doses of HU and APH were used to completely block DNA synthesis, and caffeine was used to drive Chinese hamster ovary cells (CHO) cells into mitosis, despite the presence of nonreplicated DNA. Mitotic exit was accompanied by multipolar division, thus leading to aneuploid progeny [176]. The authors showed that colcemid, but not cytochalasin, and therefore, microtubules, but not actin filaments, are critical for centrosome splitting [176]. Centrosome splitting was also observed when DNA was damaged with the crosslinking agent MMC or in repair-deficient mutants (with defective homologous recombination) [176,177,178]. Furthermore, the Sibon laboratory provided evidence that BRCA1 is recruited to centrosomes and that the absence of BRCA1 leads to centrosome number problems [179]. Damaged BRCA1-deficient cells that were forced into mitosis first showed correct bipolar establishment of the spindle, followed by failed anaphase segregation, leading to tetraploid cells with double the number of centrosomes. Subsequently, these tetraploid cells might fail to establish a bipolar spindle or efficiently cluster their centrosomes in the following mitosis.

Since failures such as centrosome splitting or overduplications are phenotypes observed after the depletion of several different repair/replication proteins; the link between hampered replication/repair and impaired centrosome integrity may be indirect. We believe that the actual presence of repair/replication proteins at centrosomes is not necessarily required; rather, the absence of these proteins during interphase creates a cell cycle delay and/or DNA damage. As synchrony between DNA replication and centriole duplication has to be guaranteed for a correct number and integrity of the centrosomes, any defect that delays the cell cycle could interfere with the centriole duplication/centrosome maturation process. Direct evidence comes from our own experiments showing that, in RAD51- or BRCA2-deficient cells, spindle multipolarity was suppressed by the simple suppression of the intrinsic replication velocity deficiencies of these cells [178,180] and that low doses of APH causes premature centriole disengagement [142].

Many experiments suggest that delaying interphase in S or G2 can lead to a continuous centrosome duplication cycle [181,182,183]. These results were mainly obtained in p53-deficient cells, and indeed, they showed that such continuous centrosome cycling is strongly suppressed in p53-proficient RPE1 cells [184], making it clear that multiple centrioles/centrosomes due to asynchrony between DNA replication and centrosome cycle can be generated but are restrained in checkpoint-proficient cells. Still, this restriction has limitations, as it was shown that continuous centriole duplication can occur in G2-arrested (RO-3306) noncancerous RPE1 cells but in the absence of obvious DNA damage or replication stress [185]. This suggests that activation of the DNA damage/DNA replication checkpoint in interphase is crucial to limiting aberrant centriole duplication cycles [184]. Concluding on the previous observations, it seems that centrioles could “in theory” continue to duplicate in the presence of DNA damage, which indeed seems to be the case for checkpoint-deficient cells, and might be limited in noncancerous cells or precancerous lesions. However, the predominant phenotype in noncancerous cells is premature mitotic centriole disengagement, which, in the very end, also leads to multipolarity during mitosis [142].

We think that centriole disengagement is a multicomponent phenomenon, where advanced centriole and centrosome maturation come together with increased microtubule stability during mitosis. Only this toxic combination drives centrioles to disengage and centrosomes to split. Both centrioles of the centrosome might, at this point, be surrounded by PCM and nucleate microtubules; in this case, microtubule forces may indeed tear the two centrioles apart. As it was shown that PCM integrity is altered after DNA damage [186], we speculate that microtubule-dependent forces destroy PCM integrity.

### 3.4. Replication Stress and Cohesion

One common idea is that centrioles, similar to sister chromatids, are held together by cohesion [187] and that both start separating at anaphase onset because of increased separase activity. Here, we summarize what is known about of the premature loss of cohesion that holds sister chromatids together. The premature loss of cohesion during mitosis is suspected to lead to faulty attachment of chromosomes due to the possibility that microtubules of opposing spindle poles attach to the same kinetochore [188]. Many studies have shown how cohesion and its correct maintenance or release impact DNA repair and replication processes [189], but less is known about how replication stress impacts cohesion maintenance in mitosis.

Under physiological conditions, WAPL opens cohesin rings, specifically at the chromosome arms, in prophase (“prophase pathway”), and centromere cohesion, protected by shugoshin, remains intact until anaphase onset, when separase cleaves it. This two-step pathway of cohesion removal, which gives metaphase chromosomes their characteristic X-shape, is crucial to maintain genetic stability [190].

Whereas we and others reported no significant increase in cohesion defects after moderate replication stress [126,142], high doses of APH and HU in p53-deficient cells led to varying degrees of cohesion defects [138]. Such defects depended on the cohesion-removing activity of WAPL, and it was suggested that a WAPL-dependent regulation of centromere cohesion is critical to achieving SAC-dependent mitotic arrest in cells challenged with lethal doses of replication stress [138]. Intriguingly, it was shown that, under sublethal APH doses, a BIR-like process in early mitosis depends on a cohesion release by WAPL [117]. Diminished cohesion is essential for the repair of damage occurring after exposure to replication stress and is one possible strategy by which cancer cells sustain proliferation after oncogene induction. In fact, many cancer cells with no mutations in cohesion subunits have adapted this strategy and show varying degrees of cohesinopathy [191].

TRF1, telomere-repeat binding factor 1, is part of the shelterin complex at telomeres and ensures correct replication at telomeres. However, it also has a largely unrecognized role in mitosis, as TRF1 depletion leads to accelerated mitotic timing, cohesion defects and merotelically attached chromosomes that induce elevated levels of n-CIN [192]. TRF1 has not been directly detected at the centromere, but its absence led to reduced levels of Aurora B and Shugoshin at centromeres and increased intercentromere distance, reminiscent of cohesion loss. Whether DNA damage signaling/replication stress that apparently is activated at telomeres after TRF1 depletion can also influence sister chromatid cohesion or whether TRF1 is directly recruited to centromeres remain open questions. Recent studies from the Gilson laboratory suggested that TRF2, but not TRF1, can be directly detected at pericentromere regions and is needed to ensure replication of the pericentromeric heterochromatin [193]. Whether TRF2 depletion elicits similar defects as TRF1 depletion and leads to increased interkinetochore distance, cohesion defects, an increased number of merotelic attachments and lagging chromosomes remains to be elucidated.

In yeast, age-related cohesion loss depends on the downregulation of cohesion subunits and is accompanied by the downregulation of DNA repair factors, which suggests that DNA damage accumulation with age is accompanied by the imperfect holding of sister chromatids together [194]. Oocytes show age-related cohesion loss [195] that might be linked to increased oxidative stress during ageing, [196,197], and oxidative stress is known to impede replication [180]. These results suggest that age-related oxidative/replication stress may be critical to the loss of sister chromatid cohesion. On the one hand, this outcome can represent a mechanism useful for eliminating “old” cells; on the other hand, failed elimination can lead to the propagation of aneuploid progeny and, eventually, cancer (cancer as an ageing condition).

**Figure 2:**
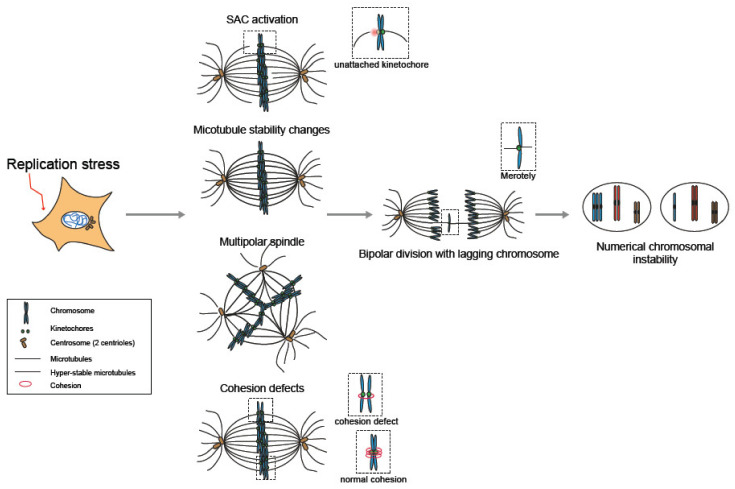
The impact of replication stress on the mitotic process. Diagram summarizing the type of mitotic dysfunctions occurring after replication stress. Replication stress can induce nonattached kinetochores and activate the spindle assembly checkpoint (SAC), change microtubule stability, induce multiple spindle poles due to centriole disengagement and lead to cohesion defects. SAC override might contribute to the mis-segregation of chromosomes with wrongly attached kinetochores. Changes in microtubule stability could lead to impaired error corrections that rely on the dynamic instability of microtubules. Multipolarity can occur transiently, because centrioles within one centrosome split before spindle poles cluster back together. Cohesion can be prematurely relieved at the centromere, which can induce chromosome mal-attachments because of altered kinetochore geometry. Wrongly attached kinetochores that are less efficiently corrected can favor merotelically attached chromatids, where microtubules of opposing spindle poles attach to the same kinetochore. Such chromatid appears as lagging in-between the two segregating DNA masses and can be either reintegrated into the correct daughter cell, integrated into the wrong daughter cell (thus leading to aneuploidy) or get enclosed by its own nuclear envelope (micronucleus).

In conclusion, it seems that the cell in the most vulnerable cell cycle phase has found quite drastic means to respond to replication stress. Even when mitotic checkpoints can partially detect replication stress, it seems that, at least, low-dose stress is transmitted through the following interphase. Furthermore, we summarized evidence that replication stress can hamper the correct bipolar spindle assembly, microtubule stability and cohesion maintenance, all of which can lead to increased levels of merotelically attached chromosomes (Figure 2), which, in a cell with 46 chromosomes, can have drastic consequences. 

## 4. How Mitotic Defects and Aneuploidy Can Lead to Replication Stress and Feed CIN to Create a Vicious Circle

**Figure 3:**
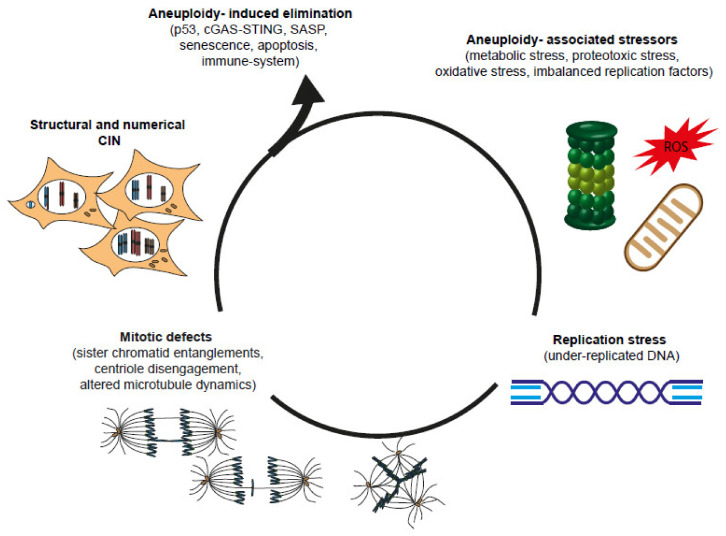
Replication stress and mitotic defects feed chromosomal instability (CIN) in a vicious circle. Diagram summarizing the vicious circle of CIN. Replication stress induced by several endogenous and exogenous sources, represented as under-replicated DNA, leads to mitotic defects (multipolar spindles in prometaphase and metaphase or lagging chromosomes, bulky and ultrafine DNA bridges in anaphase). Mitotic defects and chromosome mis-segregation will consequently lead to s- or n-CIN (structural chromosome rearrangements, micronuclei and numerical aneuploidy). Aneuploidy-associated stressors [198] (proteotoxic, metabolic, oxidative stress and altered levels of replication factors) due to gene dosage imbalance will, in turn, fuel replication stress, thus completing the CIN propagation circle. This vicious cycle can be evaded or stopped when the accumulated genomic alterations activate elimination pathways (apoptosis, senescence or immune system-mediated killing by p53, SASP or cGAS-STING), thus breaking the cycle. ROS: Reactive oxygen species.

Having considered how replication stress can lead to mitotic defects and chromosome mis-segregation, we now examine the evidence showing that mitotic defects and aneuploidy may, in turn, generate replication stress and DNA damage, thus creating a vicious circle (Figure 3).

Chromosome segregation defects may directly lead to DNA damage and structural aberrations when lagging chromosomes become trapped in the cleavage furrow, which has been shown to provoke double-strand breaks and activation of the DNA damage response [199]. Alternatively, mis-segregating chromatin and persistent anaphase bridges can induce furrow regression and tetraploidization, despite the presence of an Aurora B-dependent checkpoint that delays abscission in the presence of chromosome bridges at the cytokinetic furrow [200,201,202]. In addition, a mis-segregating chromosome incorporated in a micronucleus can undergo asynchronous and inefficient replication, leading to DNA damage and chromosome pulverization due to premature chromosome condensation [203], which may be the mechanism underlying the extensive chromosome rearrangements typical of chromothripsis [204].

In addition to the direct effects of segregation errors on genome stability, aneuploidy itself may have genome destabilizing effects [205,206]. As we explained in the previous section, mutations in genes controlling the mitotic process can severely compromise cell viability and fitness, because aneuploidy alters chromosome stoichiometry and leads to gene dosage imbalance. Various studies have highlighted that the different cellular stresses associated with aneuploidy are associated with both chromosome-specific and general effects associated with the aneuploid state [207,208,209,210]. One of the main effects of aneuploidy on cell physiology are alterations in the transcriptome and, to a lesser extent, in the proteome, which lead to an unbalanced production and assembly of multiprotein complexes. This imbalance can overwhelm the cellular systems that maintain proper protein folding and homeostasis, inducing proteotoxic stress [211], and impair specific cellular functions associated with the affected multiprotein complexes [198].

One of the main consequences linked to disturbed proteostasis is the alteration of cellular metabolism and increased production of reactive oxygen species (ROS). A higher level of ROS and altered energy metabolism have been shown in disomic yeast strains, mouse embryonic fibroblasts (MEFs) and Drosophila [212,213,214]. ROS induction in MEFs has been shown to induce oxidative DNA damage and to activate an ATM- and p53-dependent checkpoint that limits the proliferation of aneuploid cells (see below). In addition, it has been shown that aneuploidy may induce replication stress by perturbing the stoichiometry of the replication machinery, thus leading to replication fork slowing and genome instability [215]. Furthermore, replication slowing or stalling has been linked to aneuploidy-driven decreases in the expression of replication proteins [209].

### 4.1. Emerging Concepts: Chromosomal Instability/Aneuploidy Tolerance and the Immune Response

The bidirectional interplay between replication stress and chromosome mis-segregation not only promotes genome instability and transformation but may also perpetuate CIN, thus providing a mechanism of evolution for cancer cells.

Whereas replication stress and CIN play fundamental roles in driving tumor evolution, aneuploidy is known to restrain cell proliferation [216]. One of the main surveillance mechanisms arresting the proliferation of cells with abnormal karyotypes is mediated by p53 [217,218], a well-known tumor suppressor that is activated following the induction of a number of cellular stresses, including genotoxic, oncogenic and metabolic stresses [219,220].

The activation of p53 as a result of mitotic defects and aneuploidy has been attributed to different mechanisms [216], including the induction of p38 stress kinase [221], p14ARF [222], a prolonged prometaphase duration [223,224], DNA damage [199], telomere uncapping [225] and oxidative stress [214], leading to an ATM-dependent DDR. Whether chromosome mis-segregation and aneuploidy per se can induce p53 activation is still debated. An interesting study by Hinchcliff and collaborators has shown that mis-segregating chromosomes were marked along their arms by phosphorylation of the histone variant H3.3 on Ser31, which spread to both aneuploid daughter nuclei and induced p53 stabilization [226]. Another study used single-cell sequencing to show that p53 inhibited the propagation of aneuploid cells harboring structural chromosomal aberrations, while it was permissive to a subset of whole chromosome aneuploidies [227]. Similarly, another study also used single-cell sequencing to follow cell fate after chromosome mis-segregation and showed that the activation of p53 and G1 arrest occurred only in cells with highly aberrant karyotypes and was partially dependent on DDR signaling, while the majority of cells that continued to divide harbored genomic imbalances involving less than 5% of the genome [228]. Interestingly, aneuploid cells that continued to proliferate showed signs of replication stress in the following S phase and entered mitosis with under-replicated DNA, followed by the formation of UFBs and the accumulation of DNA damage, as detected by 53BP1 bodies in the next G1 phase.

These findings indicate that aneuploidy per se may not be sufficient to elicit p53 activation and that the proliferation defects of aneuploid cells may result from multiple converging stress signaling pathways [229]. In addition, aneuploidy-associated stressors, such as proteotoxic or oxidative stress, which scale with aneuploidy, can exacerbate replication stress to further induce mitotic aberrations and DNA damage [198,216,230].

Therefore, the propagation of CIN in cancer can arise through inactivation or bypass of the p53 pathway and the acquisition of mechanisms that allow tolerance of replication stress and aneuploidy-associated stressors, such as activation of the proteasome and autophagy and CIN adaptation [198,229,231]. While low and moderate levels of CIN may allow the evolution of advantageous karyotypes and the emergence of genomic alterations that provide resistance to therapy, tumors may not tolerate high CIN levels [232,233,234,235,236,237,238]. Interestingly, it has been reported that, in addition to acquiring CIN tolerance, tumors undergo CIN attenuation or buffering. One of the mechanisms enabling cells to decrease the level of CIN is by monoallelic inactivation of the anaphase promoting complex-cyclosome (APC/C) subunits [239]. Cells with decreased APC/C efficiency spend more time (approximately 10 min) in mitosis, which reduces the rate of chromosome mis-segregation. Mechanisms allowing an increase in mitotic fidelity by increasing mitotic duration include a reduced frequency of merotelic attachments [2] and centrosome clustering [240]. It would be interesting to know whether lengthening the metaphase duration may also lead to partial reactivation of p53 [241]. Another mechanism involved in CIN attenuation in tumors evolving from tetraploid cells is the loss of supernumerary centrosomes [167].

Another key brake to the proliferation of aneuploid cells is the immune system [228]. Santaguida and collaborators showed that cells with complex karyotypes induce a proinflammatory state characterized by a senescence-associated secretory phenotype (SASP), the activation of interferon alpha/beta signaling and the innate immune response through the induction of natural killer (NK) cell ligands, which renders these cells sensitive to NK-mediated elimination. Indeed, aneuploid cells were shown to activate cyclic GMP-AMP synthase-stimulator of interferon genes (cGAS-STING) signaling, an antiviral pathway that senses cytosolic DNA [228]. The activation of cGAS-STING can be associated with DNA damage-induced cellular senescence [242,243,244] due to incomplete nuclear envelope formation around micronuclei that exposes chromosomal DNA [245,246,247] or to the release of ssDNA from stalled replication forks upon replication stress [248]. Therefore, tumor cells with abnormal karyotypes must evolve mechanisms to evade the immune system.

The capacity of tumors with CIN to activate or suppress immune signaling and to drive metastasis is an emerging area in cancer biology and immunotherapy [249,250]. By analyzing the genomic data of several human tumors from The Cancer Genome Atlas, Davoli and collaborators showed that high levels of arm- and whole-chromosome somatic copy number alterations (SCNAs) were correlated with the reduced expression of markers of cytotoxic immune cell types and proinflammatory cytokines and a suppressed response to checkpoint blockade immunotherapy [251]. The immunogenic or immunosuppressive responses may also be dependent on the levels of CIN and the magnitude or duration of the immune responses [252]. Interestingly, chronic cGAS-STING activity in chromosomally unstable cancer cells has been shown to activate a noncanonical NF-κB pathway downstream of STING that promotes cell migration, invasion and metastasis [247]. In addition, cGAS has been reported to directly modulate the DNA damage response through the suppression of homologous recombination and, thus, further promotes genomic instability [253,254].

Developing new methods to detect tumor CIN, as well as therapeutic strategies based on leveraging CIN to modulate tumor fitness and immunogenicity, are therefore of utmost importance in cancer treatment [229,250].

### 4.2. Chromosomal Instability as a Double-Edged Sword in Preneoplastic and Tumor Cells

Over a century ago, the work of von Hansemann, who reported the presence of abnormal mitotic figures in tumors, and the experiments of Theodor Boveri showing the abnormal development of sea urchin eggs after dispermic fertilization, founded the hypothesis that an abnormal chromosome constitution (aneuploidy) produced by abnormal mitosis could be involved in the process of tumorigenesis (see [255]).

However, aneuploidy does not always act as a driver of tumorigenesis and, instead, is mostly detrimental for cell and organismal fitness [198,216,256]. Indeed, aneuploidy is one of the main causes of miscarriages, and very rare instances of constitutional aneuploidies compatible with life are associated with developmental disorders such as trisomy 13 (Patau syndrome), trisomy 18 (Edwards syndrome) or trisomy 21 (Down syndrome) [257]. The proliferative disadvantage associated with aneuploidy is due to the alterations in gene dosage and proteome imbalances associated with distinct metabolic changes and cellular stress [198,258,259]. The dichotomy of the role of aneuploidy in normal development and cancer [260] can be explained when comparing untransformed cells undergoing normal proliferation with cells subjected to environmental or endogenous stress, where the generation of new combinations of chromosomes allows the cells to adapt and to select variants that provide a proliferative advantage under stress conditions [229,261]. Therefore, the propensity of a cell to acquire alterations in chromosome number or structure, i.e., an ongoing CIN, a common feature of cancer [262], is also associated with developmental diseases and neurodegenerative conditions. Perhaps the best examples of how CIN and aneuploidy can lead to different outcomes are found in the studies of chromosome instability syndromes and animal models [38,263,264,265]. These studies have further revealed a relationship between aneuploidy and ageing [266,267], raising the possibility that aneuploidy is involved in tissue dysfunction and age-related pathologies [268,269].

When we discuss CIN, we have to consider it in the precancerous and cancerous contexts; although CIN is known to fuel intratumor heterogeneity, it can be detected during early tumorigenesis. Specifically, the DNA damage and chromosomal aberrations that occur in precancerous lesions as a consequence of replication stress have been shown to activate the DNA damage response that helps to eliminate damaged cells or induce them into senescence (as proposed for oncogene-induced senescence) [5,30]. However, this barrier to cell proliferation increases the selective pressure for cells harboring mutations that allows them to escape this blockade, leading to a stepwise accumulation of mutations that, in the very end, promote uncontrolled proliferation. In fully grown tumors, on the other hand, CIN is presumed to fuel genetic instability but becomes detrimental when a certain threshold is exceeded, which is extensively exploited in cancer therapy. Therefore, in each case, CIN is a double-edged sword. On the one hand, cells are prone to die, as random mutations likely do not provide any growth advantage, or die under treatment, as exacerbating an already elevated level of CIN specifically kills these cells. On the other hand, CIN harbors the risk that a cell with one mutation, which provides a growth advantage, overgrows/outgrows the rest of the population, and in both pre- and cancerous lesions, this advantage can lead to the survival and clonal expansion of this cell, which will again rapidly undergo nonclonal expansion.

As replication stress and CIN fuel cancer evolution, the problem of treating tumors that are not merely the clonal expansion of one single mutated cell but of many mutated cells becomes more complicated. Multiple mutations reduce the benefits of an initially efficient treatment, as the nonclonal expansion of cells leads to rapid adaptation to drugs. Intratumor heterogeneity not only often makes chemotherapeutic treatment ineffective but also renders the search for biomarkers, such as differently expressed proteins or tumor signatures, a race against time. As a logical consequence, targeting DNA replication, transcription or mitosis and, thus, processes that are similar for many different clones within a tumor has been revealed as a potent strategy to attenuate unrestrained proliferation.

## 5. Chromosomal Instability and Cancer Therapy

Here, we briefly discuss how cancer treatment is approached today and what we believe is missing in the current discussions. The knowledge that replication stress fuels structural and numerical chromosome aberrations simultaneously could be and should be used to target cells with intrinsic replication stress. We believe that, regardless of the underlying cause of replication stress (e.g., replication/repair deficiencies, checkpoint aberrations or metabolic stress), a common phenotype links them during mitosis, making mitosis-targeting drugs attractive investments in this context.

Classical methods to target replication stress and DNA damage include drugs that either increase DNA replication stress and/or lead to interphase DNA damage, such as cisplatin, gemcitabine, olaparib, doxorubicin or topotecan. Healthy cells should escape such treatment, as they are supposed to be not/low proliferating and/or proficient in pathways that repair the introduced DNA damage [270]. In the case of DNA double-strand breaks, the cell can repair in a rather error-prone process in G1 or in a more accurate homology-directed repair in S- and G2. In repair-deficient cells and/or cells with replication stress, introduced DNA damage and stress places additional burden on repair mechanisms. A very prominent example is the use of PARP inhibitors for BRCA1/2-deficient tumors. Nevertheless, such therapies eventually also affect healthy cells, and it has been shown that moderate stress is best suited to target tumor cells specifically [271] and for the use of combination treatments [272].

### 5.1. Microtubule-Targeting Drugs

The oldest and most efficient chemotherapeutic agent to date is paclitaxel—that is, the microtubule stabilizing compound Taxol [273], which is mainly used for breast, ovarian and lung cancer treatments. Severe side effects are neuropathies, which might arise from the action of these drugs on the interphase and their interference with microtubule-based vesicle transport or cell signaling [274]. Recently, it has been shown that intratumor concentrations of this drug do not, as believed for years, engage mitotic cell death but, rather, favor multipolar division [275].

Pioneering studies from the late 1990s demonstrated that p53 activation/stabilization after DNA damage, such as UV irradiation, bleomycin or doxorubicin, decreased Map4 (microtubule-associated protein 4) expression. Decreased expression of this microtubule binder leads to less stable microtubules and increased sensitivity to microtubule destabilizing drugs such as Vinca alkaloids [276]. Another more recent study was conducted on patient-derived xenografts (PDXs) generated from luminal or triple-negative breast cancer cells. Here, the combined inhibition of p38, which increases replication stress and/or DNA damage, and taxanes (paclitaxel and docetaxel) showed enhanced tumor responses. The authors also showed that nonresponders amongst PDXs had the least CIN at the basal level, suggesting that such a combined treatment is most beneficial in CIN^+^ tumors [277].

We discovered a synthetic lethal situation with replication stress and Taxol, which increased the number of multipolar dividing cells even when used at very low doses. Since replication stress leads to premature centriole disengagement, we propose that Taxol, which already increases multipolarity on its own, shows additive effects with APH [142]. We suspect that Taxol mainly targets centrioles that are susceptible to disengagement and microtubules that are already more stable, but we do not completely understand the mechanism. Further studies will be needed to answer the question of how APH primes cells for Taxol sensitivity. We (unpublished) and others observed that microtubule stability [278] or assembly and attachment [163] is increased in chromosomally unstable cell lines, and we provided a partial explanation of how this increased stability arises [142]. In contrast, the Bastian laboratory rescued the increased microtubule polymerization rate in CIN+ cells and in cells treated with APH by using nanomolar doses of Taxol. This treatment rescued the lagging of entire chromosomes, suggesting a synthetic viable constellation in this case [148]. Altogether, these data suggest that chromosomally unstable cells are intrinsically more vulnerable to microtubule-interfering drugs and could benefit from a combination of drugs targeting repair and/or replication in interphase and microtubules in mitosis.

### 5.2. Centrosome-Targeting Drugs

Spindle multipolarity due to centriole disengagement is the dominant and most intriguing phenotype acquired after replication stress. Indeed, we clearly observed that RPE1 noncancerous cells clustered spindle poles and ultimately divided in a bipolar manner. This data indicates an opportunity to interfere with clustering and force cells to divide in a multipolar manner, which should be detrimental. As we also observed that cells with multiple centrioles tend to disengage [142], and that many cancer cells have multiple centrioles [166], we propose that centrosome declustering drugs could be synthetically lethal when applied with replication stress. Many drugs seem to interfere with centrosome clustering [279], such as inhibitors of the motor protein HSET, Aurora A Aurora B, NEK6, PARP6 and PLK1, which provides us with a large repertoire of drugs that have been already partially established in clinics.

## 6. Conclusions

Replication stress and CIN are hallmarks of cancer that fuel each other during its evolution; however, they also represent an Achilles heel that can be exploited in cancer therapy. The identification of the molecular mechanisms underlying this bidirectional link can be useful to find biomarkers to selectively target cancer cells and to design new preventive or therapeutic strategies acting on the premitotic and postmitotic causes of CIN. The study of the molecular pathogenesis of CIN syndromes and of the mechanisms underlying their phenotypic heterogeneity will be key to informing these strategies. This knowledge can lead either to therapies attenuating the effects of replication stress in a precancerous setting or to exacerbation of the endogenous stress of cancer cells over a threshold level to induce mitotic catastrophe. Finally, the identification of CIN signatures and their relationship with the activation of inflammatory pathways and immune responses will be of increasing importance to stratify patients and select them for immune therapies.
